# Reshaping the Burden of nAMD: National, Regional, and Global Prevalence of MNV3 and Its Correlation With Life Expectancy—The RAP Study, Report 7

**DOI:** 10.1167/iovs.66.12.37

**Published:** 2025-09-17

**Authors:** Bilal Haj Najeeb, Bianca S. Gerendas, Stefan Sacu, Oliver Leingang, Gabor Deak, Ursula Schmidt-Erfurth

**Affiliations:** 1 Department of Ophthalmology, Medical University of Vienna, Austria

**Keywords:** age-related macular degeneration, macular neovascularization type 3, retinal angiomatous proliferation

## Abstract

**Background:**

To report the global distribution of macular neovascularization type 3 (MNV3) and to redefine the associated burden of neovascular age-related macular degeneration (nAMD).

**Methods:**

A total of 4652 patients with treatment-naïve nAMD were included. MNV3 was diagnosed using multimodal imaging methods. The prevalence of MNV3 in each country, region, continent and worldwide was calculated. The correlation between the prevalence of MNV3 and life expectancy in each country was estimated. Continent prevalence data was compared using a logistic (binomial) generalized linear mixed model (GLMM).

**Results:**

Our analysis revealed 4652 nAMD eyes from 47 countries, 575 (12.4%) of which had MNV3. In North America, the highest percentage was in Canada (17.6%), followed by the USA (11.5%). In Europe, the highest prevalence (23.4%) was in Austria and the lowest (4.3%) in Russia and Croatia. The prevalence in Asia, South America, and Australia was 6.6%, 9.4%, and 13.7%. A significant positive correlation was found between the MNV3 prevalence and life expectancy in 20 European countries (*r* = 0.63, *P* < 0.003). The GLMM revealed significantly lower odds for observing MNV3 in Asia compared to Europe (*P* < 0.01). No significant difference was observed in any other continent pair. The prevalence in Western Europe and Mediterranean region was higher than in Eastern Europe and the non-Mediterranean region (18.6% vs. 9.6%, *P* < 0.001; and 17.5% vs. 12.6%, *P* = 0.001, respectively).

**Conclusions:**

MNV3 is more prevalent in Western and Mediterranean regions, likely because of higher life expectancy and a lower percentage of Asian ethnicity. AREDS supplements and the Mediterranean diet may not provide prophylactic benefits against the development of MNV3. The unique distribution pattern of MNV3 and its correlation with life expectancy should be considered in nAMD research to ensure adequate representation of MNV3 patients in clinical trials.

Macular neovascularization type 3 (MNV3), also known as retinal angiomatous proliferation, is the second most common type of neovascular age-related macular degeneration (nAMD).^1^ Recent clinical studies have shown that MNV3 exhibits unique pathological, topographical, and clinical features.^2^^,^^3^ The classical clinical triad of an established lesion manifests as foveal cystoid macular edema, parafoveal intraretinal hemorrhage, and perifoveal dense exudates.^4^^–^^6^ Other characteristics have also been documented as the tendency to be bilateral, the development of retinal-choroidal anastomosis and the absence of other MNV lesions in the same eye.^6^^–^^9^ In addition, new phenotypes have also been described, such as the multifocal and the cilioretinal subtypes.^10^^,^^11^ However, epidemiological data are still scarce, demonstrating that it is more common in advanced-aged individuals and affects women more than men.^12^^–^^14^ Multiple post-hoc analyses showed a wide range in the rate of MNV3. For example, the CATT and HARBOR studies from the USA reported widely different incidence of MNV3.^15^^,^^16^ Similar variations were also observed in some studies on Asian cohorts.^17^^,^^18^ This remarkable discrepancy could be due to variations in diagnostic methods and inconsistency in the criteria used for grading MNV3.^1^ Selection bias could also be a contributing factor. For example, a considerable number of nAMD eyes could be excluded at baseline because of the presence of subfoveal hemorrhage or absence of subfoveal exudation. Furthermore, it may be explained by differences among ethnic groups.^18^^,^^19^ The need to analyze a larger number of patients across a wider geographic area prompted us to conduct this study, using large datasets and an advanced multimodal imaging approach at Vienna reading center. The study aims to evaluate the global distribution of MNV3, its correlation with life expectancy, and the potential impact on the disease burden of nAMD.

## Methods

A total of 4652 patients with newly diagnosed treatment-naïve nAMD were included in this analysis. All patients were participants in international, multicenter, prospective, randomized trials evaluating the efficacy of anti-angiogenic treatments in newly diagnosed, treatment-naïve nAMD. All studies were conducted between 2013 and 2020 with similar inclusion/exclusion criteria. No country's data were derived from a single clinical trial. Ethical approval was obtained from the Ethics Committee of the Medical University of Vienna for this analysis, and from the institution's review board of each of the participating centers for the core trials. One eye (study eye) from each patient was included. It is important to note, that to make our study as representative of real-world data as possible and to avoid any selection bias, we also included nAMD eyes that were not eligible for the corresponding main clinical trials because of study-specific exclusion criteria. For instance, nAMD eyes with subfoveal hemorrhage or atrophy, absence of subfoveal exudation (subfoveal intraretinal or subretinal fluid), or coexistence of some pathologies such as vitreomacular traction or epiretinal membrane.

The diagnosis of MNV3 was secured using a multimodal imaging approach. All eyes were classified as MNV3 or non-MNV3 based on distinguishing features in optical coherence tomography (OCT), in addition to other characteristics on color fundus photography, fluorescein angiography (FA), and elective indocyanine green angiography.^4^^,^^15^^,^^16^^,^^19^^–^^21^

The distinguishing features of MNV3 include one or more hyperreflective complexes in the outer retina appear on a disrupted pigment epithelium detachment on OCT. It is typically associated with cystoid macular edema and hard exudate, which are illustrated as prominent intraretinal fluid and accumulated hyperreflective foci, respectively. At early stages, there could be a small amount of parafoveal intraretinal fluid without clear retinal pigment epithelium disruption. On color fundus photography, a flame-shaped intraretinal hemorrhage covering the retinal neovascular complex or multiple punctate hemorrhages further away from the lesion, directed to the fovea and without subretinal hemorrhage. Another common feature is the presence of dense, hard exudate encircling the intraretinal bleeding. On FA, leakage emerges from one or more focal hyperfluorescent lesions at an abrupt curving of an enlarged retinal vessel towards the deeply situated retinal angiogenic network. It is usually accompanied by cystoid macular edema and pigment epithelium detachment on the late frames of the angiogram. On indocyanine green, one or more hot spots were associated with late-phase leakage. Also, characteristic retinal-choroidal anastomosis could be clearly illustrated.

All eyes were independently assessed by two trained and certified experienced graders, and a retina specialist acted as a judge in case of discrepancy. All sites were classified according to their geographical location for detailed analysis based on country, region, and continent. Then, we calculated the prevalence as the percentage of patients with MNV3 out of all nAMD patients for each country, region, and continent. Because this disease could be rare in certain countries or regions, 30 eyes with nAMD was considered the cutoff for calculating prevalence for each country. If the number of nAMD eyes was fewer than 30 in a given country, no national prevalence was estimated, but it was added to the corresponding region and continent calculations. Furthermore, because MNV3 is particularly known to affect advanced-aged patients, we also aimed in this analysis to calculate the linear association (correlation) between the prevalence of MNV3 and life expectancy at birth (LE) of each participating country. LE was defined as the average number of years that a newborn could expect to live if he or she were to pass through life exposed to the sex- and age-specific death rates prevailing at the time of his or her birth, for a specific year, in a given country.^22^ Accordingly, we divided Europe into Eastern and Western regions, because there is a remarkable difference in life expectancy between these two regions.^23^ In addition, we divided Europe into Mediterranean and non-Mediterranean regions to explore any association between the Mediterranean diet and the prevalence of MNV3.^24^ LE of each participating country except Taiwan was derived from the World Health Organization’s records for 2017.^22^ The correlation was determined using the Pearson correlation test. Results are reported as correlation coefficient (*r*). *P* ≤ 0.05 was considered statistically significant. Prevalence was modeled with a logistic (binomial) generalized linear mixed model (GLMM), using the country as a random factor to mitigate countries with small sample-sizes. Pairwise continent contrasts were obtained using marginal means with Tukey-adjusted *P* values.^25^^,^^26^

## Results

Our analysis revealed 4652 nAMD eyes of 4652 patients from 47 countries covering six continents. MNV3 was identified in 575 eyes, accounting for 12.4% of nAMD worldwide. All national, regional, and continental outcomes are summarized in the Table. The following are the most important outcomes:

In North America, MNV3 was evaluated in three countries with a prevalence of 11.7% (157/1344). The highest percentage was observed in Canada (17.6%), followed by the USA (11.5%). In South America, five countries were represented, but we could not calculate the frequency of MNV3 for any country because of the small size of the cohort. The prevalence of MNV3 in South America was 9.4% (5/41).

In Europe, the prevalence of MNV3 was 14.2%. 28 countries were included: 15 from Western Europe and 13 from Eastern Europe. Austria presented with the highest prevalence of MNV3 (23.4%), followed by the Netherlands (21.9%), and the lowest was seen in Russia and Croatia (4.3% each). The prevalence of MNV3 in the Western European region was statistically significantly higher than in the Eastern European region (18.6% vs. 9.6%, *P* < 0.0001) and in the Mediterranean region significantly greater than in the non-Mediterranean region (17.5% vs. 12.6%, *P* = 0.001). Using GLMM analysis, we observed that the odds of detecting MNV3 were significantly lower in Eastern compared to Western countries (odds ratio [OR] = 0.45; 95% confidence interval [CI], 0.35–0.58; *P* < 0.0001). Furthermore, patients from the Mediterranean region had significantly higher odds of MNV3 than those from the non-Mediterranean subcohort (OR = 1.50; 95% CI, 1.19–1.89; *P* < 0.001). The prevalence in Western Europe and North America was 15.1%, and in Australia it was 13.7%.

A significant moderate positive correlation was found between the prevalence of MNV3 and LE in 20 European countries (*r* = 0.63, *P* < 0.003), and also in countries with a Caucasian majority (20 European countries, the USA, Canada, Australia) (*r* = 0.62, *P* < 0.001). In Asia, MNV3 was observed in 47 of 707 (6.6%) of nAMD eyes in nine countries. The highest prevalence was noted in Israel (11.3%) and the lowest in China (2.4%). In East Asian countries (Japan, South Korea, China, Taiwan), the moderately positive correlation between the prevalence of MNV3 and LE was not statistically significant (*r* = 0.59, *P* = 0.411). Using GLMM analysis, the odds of MNV3 were significantly lower in Asia compared to Europe (OR = 0.43; 95% CI, 0.25–0.73; *P* < 0.01). After applying family-wise error correction, no other pairwise comparisons between continents reached statistical significance. The national prevalence of MNV3 in East Asian countries was much lower than that observed in countries with Caucasian majority (Europe, North America and Australia). Egypt, the only African country in our dataset, had one MNV3 case out of 10 nAMD cases.

**Table.** Prevalence of MNV3 in Patients With nAMD and LE by Country

	(MNV3/nAMD)	LE
**Europe (28**)	(358/2499) 14.2%	
**Western Europe (15)**		
Austria	(15/64) 23.4%	81.4
Netherlands	(7/32) 21.9%	81.5
* France*	(31/148) 20.9%	82.2
*Portugal*	(20/102) 19.6%	81.1
England	(31/161) 19.3%	80.9
*Italy*	(30/155) 19.3%	82.5
Germany	(32/177) 18.1%	80.8
Denmark	(6/35) 17.1%	80.9
*Spain*	(50/292) 17.1%	82.7
Switzerland	(12/87) 13.8%	83.1
Norway	(1/7)	83.9
Belgium	(1/17)	83.3
Finland	(6/10)	81.3
Ireland	(3/20)	81.6
Sweden	(0/11)	82.1
Total	(245/1318) 18.6%	
**E. Europe (13)**		
Lithuania	(6/34) 17.6%	75.5
Latvia	(9/52) 17.3%	75
*Greece*	(7/43) 16.3%	80.7
*Turkey*	(12/80) 15%	77.1
Czech Republic	(15/144) 10.4%	78.9
Poland	(14/162) 8.6%	77.6
Hungary	(27/324) 8.3%	75.9
Slovakia	(11/160) 6.9%	77.1
*Croatia*	(2/47) 4.3%	78.1
Russia	(4/94) 4.3%	72.5
Estonia	(6/22)	82.3
*Slovenia*	(0/4)	83.5
Bulgaria	(0/15)	74.7
Total	(113/1181) 9.6%	
**America (8)**		
**North America (3)**		
Canada	(9/51) 17.6%	81.7
USA	(148/1290) 11.5%	78.5
Mexico	(0/3)	75.9
Total	(157/1344) 11.7%	
**South America (5)**		
Argentina	(2/9)	76.6
Paraguay	(0/4)	75.3
Guatemala	(1/12)	76.6
Brazil	(0/3)	75
Colombia	(2/13)	77.8
Total	(5/41) 9.4%	
**Asia (9)**		
Israel	(18/160)11.3%	82.4
S. Korea	(13/119) 10.9%	83.1
Taiwan	(2/33) 6.1%	80
Japan	(9/186) 4.8%	84.3
China	(4/168) 2.4%	76.9
Vietnam	(0/12)	73.4
India	(0/11)	70.1
Malaysia	(0/1)	74.4
Singapore	(1/17)	83.2
Total	(47/707) 6.6%	
**Oceania (1)**		
Australia	(7/51)13.7%	82.7
Total	(7/51) 13.7%	
**Africa (1)**		
Egypt	(1/10)	71.3
Total	(1/10)	
All countries	(575/4652) 12.4%	

Prevalence is listed in descending order within each region/continent. MNV3 prevalence (%) was calculated only for countries with ≥30 nAMD cohorts. European Mediterranean countries are italicized.

## Discussion

This study has shown that MNV3 accounts for about 12.4% of nAMD cases globally. Remarkably, it is not equally distributed across the world. While it is more common in regions/countries of higher LE such as Western Europe and Canada, it is much less prevalent in Asia and regions/countries of lower LE such as Eastern Europe and China.

In the USA, 11.5% of nAMD patients had MNV3. Previous post-hoc analyses of the CATT and HARBOR trials found that 10.7% (126/1183)^16^ and 21% (15/700)^15^ of nAMD patients had MNV3, respectively. The high percentage observed in the HARBOR study could be explained by the introduction of OCT, which is more accurate in diagnosing MNV3 compared to FA alone, as used in the CATT study. Furthermore, this could be caused by including American sites of varying LE. Given that MNV3 particularly affects advanced-aged patients, the latter explanation is also supported by a study conducted at a private retina practice in New York, USA, on patients with a higher mean age (86.3 years; six and eight years older than those of patients of the CATT and HARBOR studies, respectively),^16^^,^^27^ where MNV3 accounted for 34.2% of nAMD patients.^28^ Likewise, at a private practice in Valencia, Spain, a city with a high LE similar to New York, MNV3 was identified in about one third of nAMD cases.^29^ Thus an increase in the LE of a population, reflected in the higher mean age of the recruited nAMD cohorts, leads to a higher prevalence of MNV3. This could explain, for example, the higher prevalence in Canada compared to the USA, and in Western Europe compared to Eastern Europe in our analysis.

In the same context, an interesting study found that MNV3 did not appear in age group of 50–60 years, but its prevalence gradually increased with age, reaching 7.5% in nAMD patients aged 60–70, 16.8% in patients aged 70–80, and 26.5% in those aged 80 or older.^30^ Given that MNV3 is not preceded, coexistent or followed by other MNV types, this suggests that other types are less prevalent in advanced age and vice versa.^13^^,^^31^

This trend in MNV3 could help nAMD investigators to preferentially select advanced-aged patients if they seek faster recruitment and meaningful appearance of MNV3 in their studies. It also recalls our question about whether the outcomes of the AREDS study, which investigated the prophylactic effects of AREDS supplements against the development of late AMD, are also valid for eyes that will develop MNV3, given that half of the recruited late AMD-free cohorts in AREDS were 68 years or younger at baseline.^32^^,^^33^

Our study also revealed a positive correlation between MNV3 prevalence and LE in Asian countries, but it was statistically underpowered, mainly because only four countries recruited ≥30 nAMD cohorts. The prevalence of MNV3 in several Asian countries in our study is comparable to previous studies (2% in China, 6.1% in Japan, and 13.6% in South Korea).^13^^,^^34^^,^^35^ These studies have also proven that Asians with MNV3, similar to Caucasians, are advanced in age.^36^ Some of these studies included a larger number of cohorts than ours, indicating the real-world nature of our study.^11^^,^^15^^,^^16^

Of note, the prevalence of MNV3 in Asia was much lower than that observed in patients of European ancestry (Europe, North America, Australia). This is because PCV is much more frequent in Asians, accounting for more than half of new nAMD cases^37^ compared to 9% in Caucasians.^38^^,^^39^

Interestingly, MNV types were classified in 1600 nAMD Japanese patients over 10 years. The researchers, who divided the study chronologically into five 2-year periods (phases 1–5),^13^ observed an increase in the incidence of MNV3 during the last two phases (four years). Consequently, we conducted a subgroup analysis based on their published raw data, where we found a statistically significant increase in the mean age of cohorts in the last four years compared to the first six years (75.1 ± 8.1 vs. 73.5 ± 8.7, *P* < 0.0001) (unpublished data). In addition, the distribution of MNV3 patients among age groups was as follows: 0% in the 50–60 age group, 10% in the 60–69 age group, 25% in the 70–79 age group, and 65% in patients aged 80 years or older (unpublished data). These outcomes reinforce our observation that higher LE is associated with a greater prevalence of MNV3. Accordingly, the increase in the prevalence of MNV3 from 10% in Western Europe and North America in the VIP study in 1998 to 15.1% in our study could also be largely related to the increase in LE in these countries over about 20 years.^40^

The nAMD is expected to remain a major cause of blindness in the elderly.^41^^,^^42^ However, the trend observed in our analysis suggests that the frequency of MNV3 will particularly increase due to the exponential aging of the population. Thus, when counted by eyes (not patients), MNV3 may replace MNV1 as the most common MNV type in Caucasians, because MNV3 is much more likely to be bilateral than other types.^9^ Such a projection could represent an important public health burden in countries with high LE, given that MNV3 is associated with poor visual function and is more exudative and recurrent than MNV1.^5^^,^^43^

Western Europe had the highest prevalence of MNV3 worldwide. For example, in Austria, MNV3 accounted for about a quarter of new nAMD cases, followed by the Netherlands. Such a high rate has been observed in a recent Austrian study,^44^ whereas Russia and Croatia in East Europe exhibited the lowest prevalence.

Many recent studies have reported on the prophylactic role of the Mediterranean diet in preventing the development of late AMD.^24^^,^^45^ However, the prevalence of MNV3 was statistically significantly higher in Mediterranean countries than in non-Mediterranean ones. This could refer that the prophylactic effect of the Mediterranean diet may be weaker in individuals who will develop MNV3 compared to those who will develop MNV1 or MNV2.

Even though earlier AMD studies identified either no or weak association with gender,^46^^,^^47^ recent studies have shown that MNV3 is more prevalent in women than in men without providing a clear explanation.^14^^,^^16^^,^^37^^,^^48^^,^^49^ Our observation that MNV3 prevalence correlates with LE could be a possible reason, as women live longer than men worldwide.^22^

This report has some limitations. First, no demographic characteristics of our cohorts such as age, gender or ethnicity were evaluated. This intentional masking was a mandatory procedure to ensure an independent grading of all prospective, international, randomized clinical studies at Vienna reading center. Second, many participating countries recruited only a small number of individuals, making some national prevalence calculations impossible. However, the robust outcomes from the remaining countries allowed us to draw meaningful conclusions. Third, because the datasets were derived not from population-based epidemiological studies but from various clinical studies, there could be significant potential for selection bias, which may limit the generalizability of our cohort to the real-world nAMD population. Nonetheless, conducting an optimal global epidemiological study with advanced multimodal imaging methods would require huge personnel resources and financial expenditure. Consequently, as mentioned in the Methods section, to make our analysis as representative of the real-world conditions as possible, we included nAMD patients who had been excluded from these core clinical trials due to specific exclusion criteria. Fourth, the correlation between MNV3 prevalence and life expectancy should be interpreted with caution in countries with low life expectancy (i.e., less than 70 years), as these countries were underrepresented in our study. However, even in real-world data, reliable prevalence estimates—both for MNV3 and for nAMD—are largely lacking in many of these regions, particularly in African and Central Asian countries.

In conclusion, this is a novel analysis estimating the national, regional and global prevalence of MNV3 using standardized accurate diagnostic methods. MNV3 is responsible for one of eight new nAMD cases globally. It is not evenly distributed across the globe. Asian ethnicity and life expectancy are crucial determinants for its frequency in each country or region. Given that MNV3 prevalence will likely increase because of the growing number of advanced-aged individuals worldwide, this study also highlights the need for a strategy to optimize the management of this highly exudative type of MNV.
